# Laparoscopic Retroperitoneal Nephron-Sparing Surgery Without Renal Artery Clamping with Preoperative Selective Arterial Embolization for Management of Right Renal Angiomyolipoma of Diameter 10 cm: A Case Report

**DOI:** 10.1089/cren.2016.0133

**Published:** 2017-02-01

**Authors:** Tatsuhiko Hoshii, Shinichi Morita, Yohei Ikeda, Go Hasegawa, Tsutomu Nishiyama

**Affiliations:** ^1^ Department of Urology, Niigata University Medical and Dental Hospital, Niigata, Japan.; ^2^ Department of Gastroenterology, Niigata University Medical and Dental Hospital, Niigata, Japan.; ^3^ Department of Diagnostic Radiology, Niigata University Medical and Dental Hospital, Niigata, Japan.; ^4^ Pathology, Uonuma Institute of Community Medicine, Niigata University Medical and Dental Hospital, Niigata, Japan.

**Keywords:** kidney, angiomyolipoma, laparoscopic surgery, nephron-sparing surgery, arterial embolization

## Abstract

A 38-year-old female without the tuberous sclerosis complex was diagnosed with right renal angiomyolipoma of 10 cm in diameter. She underwent laparoscopic retroperitoneal nephron-sparing surgery without renal artery clamping with preoperative selective arterial embolization to avoid a significant risk of hemorrhage and the damage of the renal function during nephron-sparing surgery. The tumor was resected completely. The time taken to complete the procedure was 4 hours 11 minutes and blood loss was 780 mL. She was transfused 400 mL of autologous blood.

## Introduction

Renal angiomyolipoma (AML) is an uncommon benign tumor that can occur sporadically or as a manifestation of tuberous sclerosis complex or sporadic lymphangioleiomyomatosis.^1^ The majority of AMLs are often asymptomatic and detected incidentally, with hemorrhagic presentation being less common. For management of AML, active surveillance remains the first-line option in properly asymptomatic cases involving small AMLs. Treatment might be necessary in symptomatic presentations or when the mass exceeds 4 cm in size. Nephron-sparing surgery and selective arterial embolization are preferred management strategies in cases involving symptomatic or large tumors due to increased risk of hemorrhage. Each option may have benefits, depending on the clinical situation.

We report a case of laparoscopic retroperitoneal nephron-sparing surgery without renal artery clamping with preoperative selective arterial embolization for the management of right renal AML of 10 cm in diameter to avoid a significant risk of hemorrhage and the damage of the renal function during nephron-sparing surgery with an excellent result.

## A Case Report

**FIG. 1.**
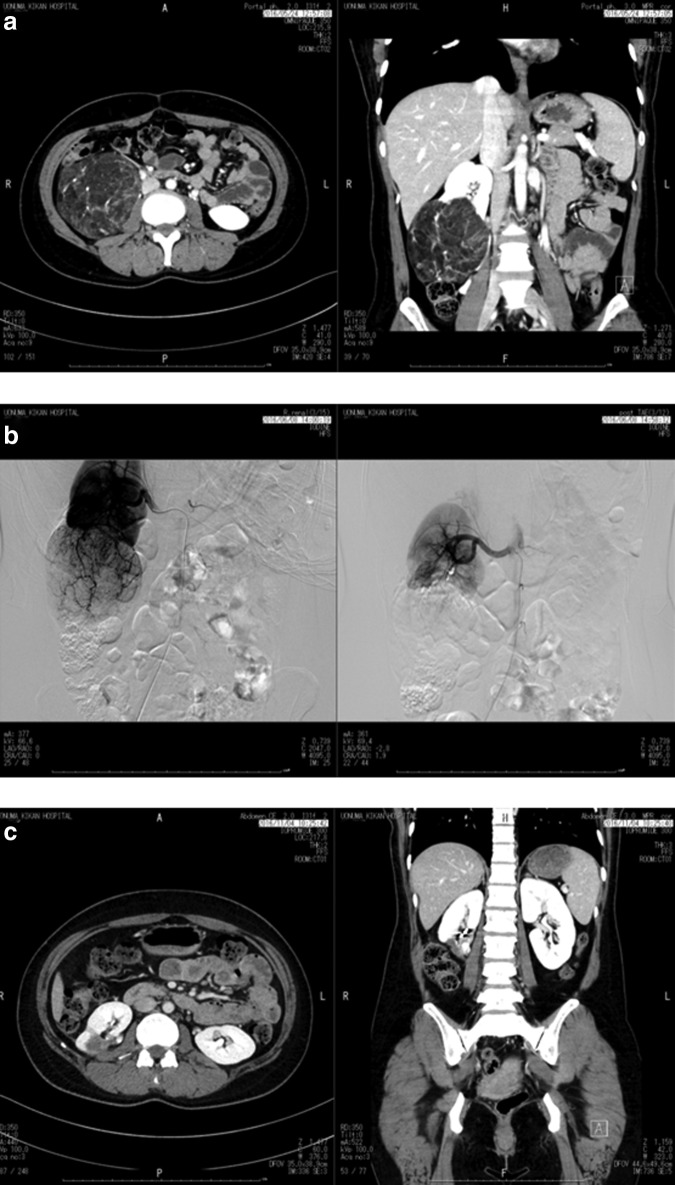
**(a)** The transverse and coronal enhanced CT images show a right renal mass mainly containing fat. **(b)** The digital subtraction angiography images. The pre-embolization image (*left*) shows a right renal hypervascular mass. The postembolization image (*right*) shows disappearance of tumor stain. **(c)** The postoperative CT images show a postoperative change in the right kidney.

A 38-year-old female without the tuberous sclerosis complex was diagnosed with right renal AML of diameter 4 cm in her health checkup in March 2010. However, she was not recommended further examination and treatment. In December 2015, she was diagnosed with right renal AML of diameter 9 cm in her health checkup and recommended management of the tumor. She visited a certain hospital for a second opinion and was recommended open right partial nephrectomy. She desired more minimally invasive treatment and visited our hospital for further opinion in March 2016. We recommended laparoscopic retroperitoneal nephron-sparing surgery without renal artery clamping with preoperative selective arterial embolization for the management of her right renal AML. She chose our therapeutic strategy. The tumor attached to lower side of right kidney of diameter 10 cm (Fig. 1a). Four hundred milliliters of preoperative autologous blood was collected from the patient. She was treated with transcatheter arterial embolization of tumor feeding artery in June 2016 (Fig. 1b). She underwent three-dimensional laparoscopic retroperitoneal nephron-sparing surgery without renal artery clamping on the day after the embolization.

## Operative Findings

**FIG. 2.**
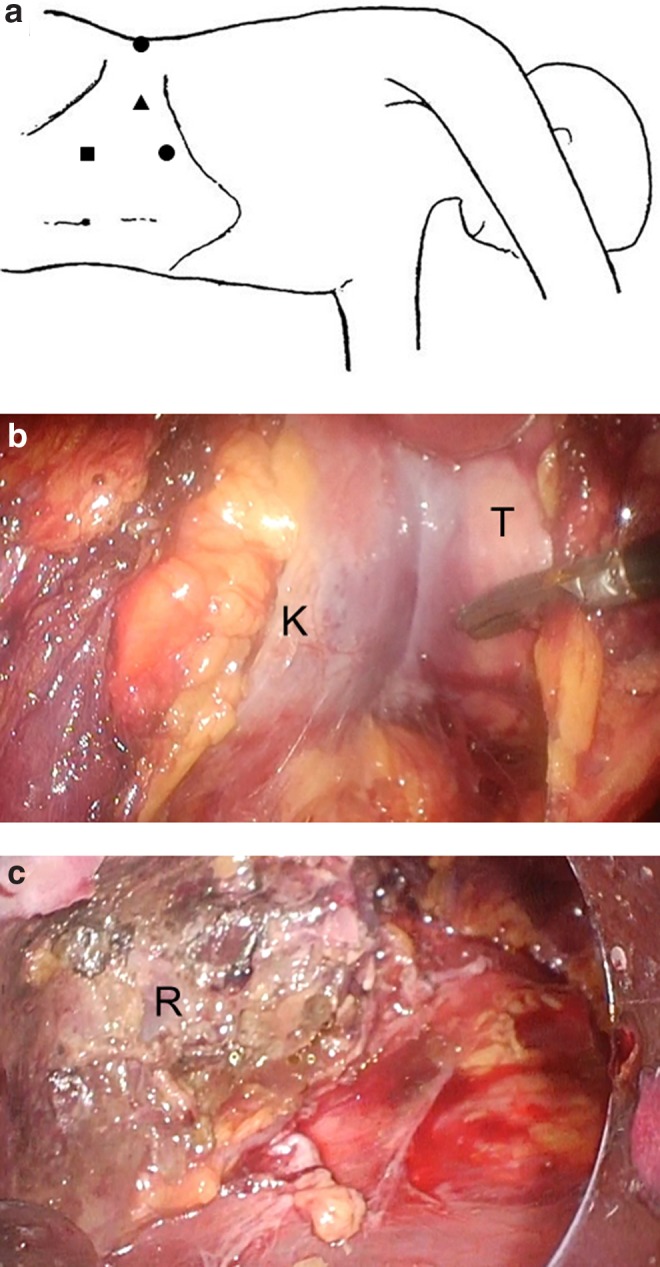
The placement of the trocar ports. ●, operator's ports. ▲, camera port. ■, assistant's port. **(a)** Laparoscopic view of right kidney and the tumor. K, right kidney, T, tumor. The boundary line between normal renal parenchyma and tumor is constricted and well circumscribed. **(b)** Laparoscopic view of the resection plane of right kidney after removal of the tumor. R, resection plane. The tumor is resected completely from normal renal parenchyma.

Right retroperitoneal space was created and four operating ports were placed (Fig. 2a). There was hardly any adherent with the surrounding tissues following the influence of preoperative selective arterial embolization. Operatively, the tumor was located at the lower side of the kidney. The boundary line between normal renal parenchyma and tumor was constricted and well circumscribed (Fig. 2b). The tumor was exfoliated and separated from normal renal parenchyma. The tumor was resected completely (Fig. 2c). The tumor was placed into the organ collecting bag and chopped into small pieces in the organ collecting bag and extirpated out of the body. The collecting system was confirmed to be intact after removal of the tumor using retrograde indigo carmine infusion to right renal pelvis.

The time taken to complete the procedure was 4 hours 11 minutes and blood loss was 780 mL. She was transfused 400 mL of autologous blood.

## Pathological Findings

**FIG. 3.**
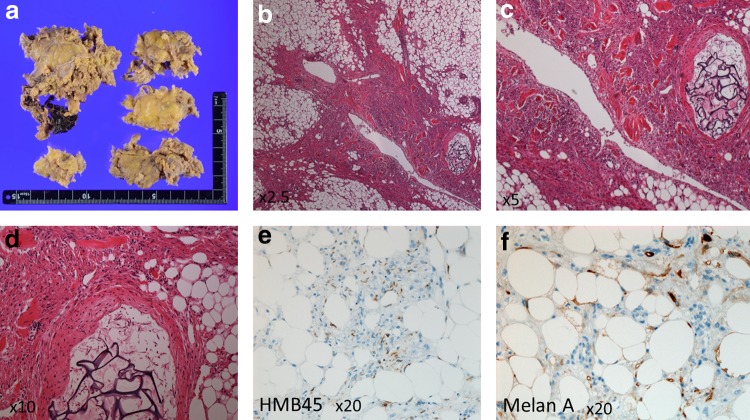
Macroscopy **(a)**: a pale yellow color tumor tissue with coagula. Microscopy **(b–f)**: histologically, the tumor is characteristically admixed and composed of blood vessels, smooth muscles, and adipose tissues, and adipose tissue component was inconspicuous **(b-d:** hematoxylin and eosin staining**)**. The smooth muscles are often associated with the muscle layer of the abnormal-looking blood vessels. In the space of small artery, there are materials of the transcatheter arterial embolization. Immunohistochemical examination of melanocytic markers; the tumor cells are positive for HMB45 **(e)** and MelanA **(f)**.

Histopathology showed that the tumor was characteristically admixed and composed of blood vessels, smooth muscles, and adipose tissues (Fig. 3).

## Postoperative Findings

The patient's postoperative course was uneventful. Postoperative first day, the drain was removed and she was discharged from the hospital on postoperative day 5. Four months later, the right kidney was a normal except postoperative defect and scar formation (Fig. 1c).

## Discussion

For the management of AML, active surveillance remains the first-line option in properly asymptomatic cases involving small AMLs.^1,2^ Treatment might be necessary in symptomatic presentations or when the mass exceeds 4 cm in size. Options for treatment have traditionally included radical and partial nephrectomy, selective arterial embolization, and ablative therapies, including cryoablation and radiofrequency ablation. Each option may have benefits, depending on the clinical situation; however, there are no randomized trials that have compared these approaches. The level of evidence in the current literature does not allow a recommendation of a particular option over another. Nephron-sparing surgery and selective arterial embolization are preferred management strategies in cases involving symptomatic or large tumors due to increased risk of hemorrhage.^1,3^ Selective arterial embolization is effective in controlling hemorrhage from AMLs in the acute setting. Selective arterial embolization is a good treatment method; however, repeat embolization or surgery was required in over 20% following the embolization.^3^ Selective renal artery embolization may be used when the size or location precludes nephron-sparing surgery.

Prophylactic surgery is generally indicated to prevent hemorrhage among patients with renal AMLs larger than 4 cm in diameter.^1^ Nephron-sparing surgery is preferred rather than complete nephrectomy for patients with AMLs who are selected for intervention. Previously, nephron-sparing surgeries were performed in open surgery; however, recently many of nephron-sparing surgery were performed laparoscopically and laparoscopic nephron-sparing surgery is considerably less invasive.^4^ Laparoscopic partial nephrectomy for large renal mass requires long operative duration. Prolonged renal artery clumping for nephron-sparing surgery reduces kidney function. To preserve kidney function, nephron-sparing surgery without clumping of renal artery is a risk of massive bleeding. We performed laparoscopic retroperitoneal nephron-sparing surgery without renal artery clamping with preoperative selective arterial embolization for the management of right renal AML of 10 cm in diameter to avoid a significant risk of hemorrhage and the damage of the renal function during nephron-sparing surgery with an excellent result. It was difficult to identify the feeding artery to the tumor in the operation in this case. If we performed partial nephrectomy without renal artery clamping and preoperative selective arterial embolization, we could have suffered from massive bleeding during operation. Most of the 780 mL blood loss in this case was from venous back flow and not from arterial bleeding. Therefore, the blood loss was thought to be not able to be so decreased with clumping of the renal artery.

Taking into consideration, the reduction of kidney function due to renal artery clumping to avoid the risk of massive bleeding during operation, laparoscopic nephron-sparing surgery without renal artery clamping in combination with preoperative selective arterial embolization is a good way of treatment-needed renal AML to avoid a significant risk of massive hemorrhage and the damage of the renal function during nephron-sparing surgery.

## Abbreviations Used

AML: angiomyolipoma
CT: computed tomography

## References

[1] Flum AS, Hamoui N, Said MA, Yang XJ, Casalino DD, McGuire BB, Perry KT, Nadler RB Update on the diagnosis and management of renal angiomyolipoma. J Urol 2016;195(4P1):834–846 26612197 10.1016/j.juro.2015.07.126

[2] Sooriakumaran P, Gibbs P, Coughlin G, et al. Angiomyolipomata: Challenges, solutions, and future prospects based on over 100 cases treated. BJU Int 2010;105:101–106 19493268 10.1111/j.1464-410X.2009.08649.x

[3] Murray TE, Doyle F, Lee M Transarterial embolization of angiomyolipoma: A systematic review. J Urol 2015;194:635–639 25916674 10.1016/j.juro.2015.04.081

[4] Msezane L, Chang A, Shikanov S, Deklaj T, Katz MH, Shalhav AL, Lifshitz DA Laparoscopic nephron-sparing surgery in the management of angiomyolipoma: A single center experience. J Endourol 2010;24:583–587 20423289 10.1089/end.2009.0330

